# Association of Intensive Care Unit Case Volume With Mortality and Cost in Sepsis Based on a Japanese Nationwide Medical Claims Database Study

**DOI:** 10.7759/cureus.65697

**Published:** 2024-07-29

**Authors:** Takehiko Oami, Taro Imaeda, Taka‑aki Nakada, Tuerxun Aizimu, Nozomi Takahashi, Toshikazu Abe, Yasuo Yamao, Satoshi Nakagawa, Hiroshi Ogura, Nobuaki Shime, Yutaka Umemura, Asako Matsushima, Kiyohide Fushimi

**Affiliations:** 1 Department of Emergency and Critical Care Medicine, Chiba University Graduate School of Medicine, Chiba, JPN; 2 Health Services Research and Development Center, University of Tsukuba, Tsukuba, JPN; 3 Critical Care Medicine, National Center for Child Health and Development, Tokyo, JPN; 4 Trauma and Surgical Critical Care, Osaka General Medical Center, Osaka, JPN; 5 Department of Emergency and Critical Care Medicine, Graduate School of Biomedical and Health Sciences, Hiroshima University, Hiroshima, JPN; 6 Emergency Medicine, Osaka General Medical Center, Osaka, JPN; 7 Department of Emergency, Nagoya City University East Medical Center, Nagoya, JPN; 8 Department of Health Policy and Informatics, Tokyo Medical and Dental University, Tokyo, JPN

**Keywords:** sepsis, medical cost, hospital case volume, diagnosis procedure combination, critical care

## Abstract

Background

The impact of intensive care unit (ICU) case volume on the mortality and medical costs of sepsis has not been fully elucidated. We hypothesized that ICU case volume is associated with mortality and medical costs in patients with sepsis in Japan.

Methodology

This retrospective nationwide study used the Japanese administrative data from 2010 to 2017. The ICU volume categorization into quartiles was performed according to the annual number of sepsis cases. The primary and secondary outcomes were in-hospital mortality and medical costs, respectively. A mixed-effects logistic model with a two-level hierarchical structure was used to adjust for baseline imbalances. Fractional polynomials were investigated to determine the significance of the association between hospital volume and clinical outcomes. Subgroup and sensitivity analyses were performed for the primary outcome.

Results

Among 317,365 sepsis patients from 532 hospitals, the crude in-hospital mortality was 26.0% and 21.4% in the lowest and highest quartile of sepsis volume, respectively. After adjustment for confounding factors, in-hospital mortality in the highest quartile was significantly lower than that of the lowest quartile (odds ratio = 0.829; 95% confidence interval = 0.794-0.865; p < 0.001). Investigations with fractional polynomials revealed that sepsis caseload was significantly associated with in-hospital mortality. The highest quartile had higher daily medical costs per person compared to the lowest quartile. Subgroup analyses showed that high-volume ICUs with patients undergoing mechanical ventilation, vasopressor therapy, and renal replacement therapy had a significantly low in-hospital mortality. The sensitivity analysis, excluding patients who were transferred to other hospitals, demonstrated a result consistent with that of the primary test.

Conclusions

This nationwide study using the medical claims database suggested that a higher ICU case volume is associated with lower in-hospital mortality and higher daily medical costs per person in patients with sepsis.

## Introduction

Sepsis affects millions of patients due to its associated lethality, persistent organ dysfunction, and impaired quality of life, making it a public concern worldwide [1,2]. To address the emerging situation of an increasing number of patients with sepsis over the years, centralization of patients in high-volume hospitals might be the efficient strategy to improve patient outcomes through abundant medical resources and high capability in implementing protocol-based care [3,4]. In the field of surgery and trauma, high-volume hospitals are associated with decreased mortality rate, low frequency of surgical complications, and shorter length of hospital stay [5,6]. In accordance with these benefits, centralization of patients has been regarded as an essential strategy for achieving positive outcomes in certain situations.

Concerning sepsis cases, a previous meta-analysis of 10 observational studies found a dose-dependent association between high-volume hospitals and increased survival [7]. As patients with sepsis require a substantial amount of medical resources, including specialized staff, therapeutic interventions, and intensive care management, the impact of hospital case volume should be investigated primarily in sepsis cases admitted to the intensive care unit (ICU). Although the need for ICUs is increasingly recognized, the debate on the optimal size and case volume of these units remains unresolved [8-14]. Notably, research from Asia, and Japan, in particular, is scarce in this domain, indicating a gap that this study aimed to fill.

The few studies regarding sepsis case volumes scarcely addressed the economic implications, whereas the relationship between case volume and medical costs has been presented in the fields of surgery, trauma, and burn injury [5,15,16]. Given the direct relationship between treatment intensity, resource use, and healthcare spending [17], the impact of sepsis case volume on both costs and mortality rates warrants exploration. The perspective on healthcare costs in sepsis is expected to add a novel dimension to the discourse. Therefore, using a Japanese nationwide medical claims database, we tested the hypothesis that ICU case volume is associated with mortality rate and medical costs in patients with sepsis. This study aimed to contribute to this discussion by providing insights specific to the Japanese healthcare context.

## Materials and methods

Study setting and patient data

This retrospective study used the Japanese nationwide medical claims database known as the Diagnosis Procedure Combination (DPC) [18]. The DPC data were obtained from nearly all acute care facilities, including teaching hospitals and critical care centers that have been authorized by the Ministry of Health, in 2017. Although DPC has a code of sepsis, it is not based on the diagnostic criteria, which may result in low diagnostic accuracy. As such, we identified patients with sepsis through a screening process that incorporated a combined diagnosis of presumed serious infection and organ dysfunction, as described in a previous report [19-23], which aligned with the diagnostic criteria of Sepsis-3 [1]. Presumed serious infection was defined by four consecutive days of antibiotic administration with a blood culture drawn 48 hours before or after the first dose of antibiotics [20]. Patients who either died or were transferred to other hospitals before completing the full four-day duration of antibiotic administration were also included in the study. As laboratory data were not recorded retrospectively in this database, organ dysfunction was determined based on other integrated conditions such as vasopressor use, oxygen support, ventilation support (including non-invasive mechanical ventilation), renal replacement therapy (RRT), or diagnostic codes related to organ dysfunction. Patients with end-stage renal disease on maintenance dialysis or under the age of 20 were excluded from this study. We used the same database in the previous reports [20-23]. The ICU case volume categorization into quartiles was estimated by the annual number of sepsis cases admitted to the ICU. We calculated the annual ICU case volume with the average number of patients with sepsis for eight years. To remove the outliers, we applied the threshold of 2% of top and bottom of the number of sepsis cases according to the previous study [24].

The Institutional Review Board of Chiba University Graduate School of Medicine approved the study (approval number: 3429) and waived the requirement for written consent due to the retrospective nature of this study in accordance with the Ethical Guidelines for Medical and Health Research Involving Human Subjects in Japan. This article was previously posted to the Research Square preprint server on October 11, 2022 [25].

Data collection

The following patient demographic data and hospital characteristics were collected from the database: age, sex, chronic diseases, Barthel Index, medical procedures, medical costs, length of ICU stay, length of hospital stay, site of infection, number of hospital beds, and academic affiliation. Admission diagnosis, comorbidities, or complications during the hospital stay were recorded as codes based on the International Statistical Classification of Diseases and Related Health Problems 10th revision (Table 1). Patients with multiple codes for the “site of infection” parameter were categorized as “multiple,” whereas those with missing data (n = 161,451) were categorized as “unknown.” In the case of repeat admissions, the only last admission was included in the analysis. Patients who were admitted to more than one hospital for sepsis during the study period were not distinguished in this analysis. Patients whose cultures or antibiotics were initiated within 48 hours of hospital admission were defined as having community-acquired sepsis.

**Table 1:** Diagnostic categories with corresponding International Statistical Classification of Diseases and Related Health Problems 10th revision (ICD-10) codes.

Diagnosis	ICD-10 codes
Comorbidity	
Malignant tumor	C00-C97, D00-D09
Hypertension	I10-I15
Diabetes mellitus	E10-E14
Heart failure	I50
Ischemic heart disease	I60-I69
Cerebrovascular disease	I20-I25
Chronic respiratory disease	J40-J47
Chronic renal failure	N18
Focus of infection	
Respiratory	A15-A16, J00-J06, J09-J18, J20-J22, J31-J32, J35-J37, J39.0, J39.1, J85-J86
Urogenital	A18.1, A51.0, A54.0-A54.2, A56.0-A56.2, A59.0, A60.0, N30.0, N30.8, N39.0, N41.0-N41.3, N45, N49.0-N49.2, N70-N77, O23
Abdominal	A00-A09, A18.3, A42.1, A74.8, K35-K38, K57.0, K57.2, K57.4, K57.8, K61, K63.0, K63.1, K65, K67, K75.0, K80.0, K80.1, K80.3, K80.4, K81, K83.0
Bone and soft tissue	A18.0, A18.4, A26.0, A28.1, A31.1, A31.8, A32.0, A36.3, A42.2, A43.1, A46, A48.0, L00-L08, M00, M01.0, M46.3, M46.5, M49.1-M49.3, M60.0, M86.0, M86.1, M86.65, M86.66, M86.69, M86.99
Blood	A19, A40.0, A49.0, A49.1, A49.9
Organ dysfunction	
Renal	N00.9, N10, N17.0, N17.1, N17.8, N17.9
Hepatic	K72.0, K72.9, K76.8
Thrombocytopenia	D69.5, D69.6
Coagulopathy	D65, D68.9
Acidosis	E87.2

Medical costs

The total medical costs, including the medication fees, procedures, examinations, radiology, and hospitalization, were calculated according to the reference prices in the Japanese fee schedule for each year, as described in a previous report [21]. In the DPC system, the determination of medical fee reimbursement is primarily based on the principal diagnosis, which represents the major utilization of medical resources, as well as the medical procedures conducted during the hospitalization. As this bundled payment system does not reflect the severity of the patient’s illness, we summed the individual fees for each patient in this study. The value of medical costs was standardized by the consumer index price each year. After normalization, the price was converted from Japanese yen to U.S. dollars as of January 11, 2023 (132.47 yen = 1 USD).

Statistical analysis

In this study, the primary outcome was the in-hospital mortality according to the annual number of sepsis patients. The secondary outcomes were medical costs and length of hospital stay. In this study, we used both continuous and categorical variables to analyze the hospital volume [11]. We used a mixed-effects logistic model with a two-level hierarchical structure, including hospital-level clustering, to adjust for the baseline imbalances as follows: the independent variable was adjusted for age, sex, site of infection, community-acquired sepsis, chronic diseases, Barthel Index, vasopressor therapy at the time of sepsis onset, mechanical ventilation at the time of sepsis onset, RRT at the time of sepsis onset, admission year, number of hospital beds, and academic affiliation. As the severity scores using laboratory tests were not available in our study, we used organ dysfunctions and therapeutic interventions at the time of sepsis onset to estimate the extent of the severity [22,23,26]. According to the definition that sepsis patients in our study required the administration of antibiotics within 48 hours before or after blood culture collection, we identified patients who received vasopressor therapy, oxygen therapy, mechanical ventilation, or RRT within two days of blood culture collection as variables to assess cardiovascular, respiratory, and renal dysfunction.

In the analysis using the number of admitted patients as a continuous variable, fractional polynomials were used to determine the significance of the association between the hospital volume and clinical outcomes. For sensitivity analysis, we estimated a model with restricted cubic splines to examine the correlation in the case of the non-linear association between the number of admitted patients and the mortality, length of hospital stay, and medical costs. To determine the optimal number of knots in the restricted cubic spline, we compared Akaike’s information criterion (AIC) and Bayesian information criterion (BIC) among different models. Moreover, we analyzed the cohort after excluding patients who were transferred to other hospitals and after applying a different threshold with the top and bottom of 5% of the annual number of patients with sepsis. Furthermore, we conducted subgroup analyses concerning patients undergoing mechanical ventilation, vasopressor therapy, and RRT to validate the results in a specific population.

Categorical variables are expressed as numbers and percentages. These values were analyzed using Pearson’s chi-square test. Continuous variables are expressed as means (standard deviation) or medians (interquartile ranges (IQR)), as appropriate. The use of the statistical thresholds, as determined by the p-value, was applied for the primary outcome only. Data were manipulated and analyzed using SQL (MariaDB v10.4.17), R version 4.1.2 (R Foundation for Statistical Computing, Vienna, Austria), Stata version 17.0 (StataCorp LLC, College Station, TX, USA)), and pandas (v1.0.5), scipy (v1.7.3), numpy (v1.21.4), seaborn (v0.11.2), matplotlib (v3.5.1), and statsmodels (v0.13.2) in Python (v3.9.0).

## Results

Baseline patient characteristics

**Figure 1:**
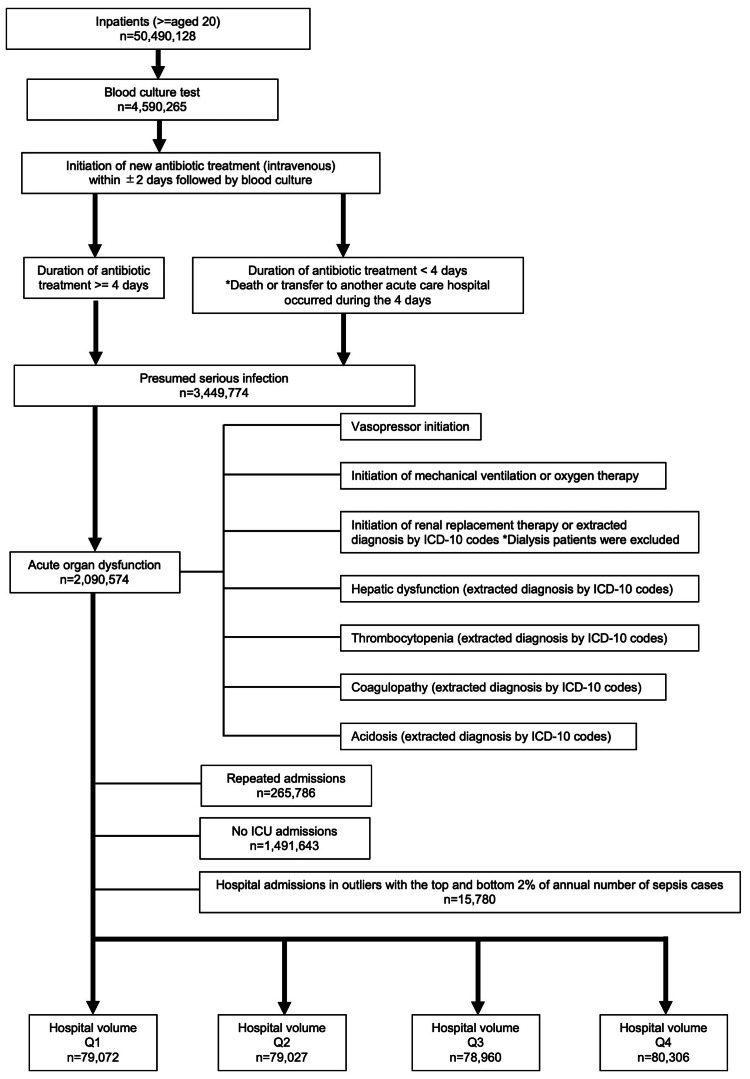
Flowchart of the study population. ICD-10 = International Statistical Classification of Diseases and Related Health Problems 10th revision

**Figure 2:**
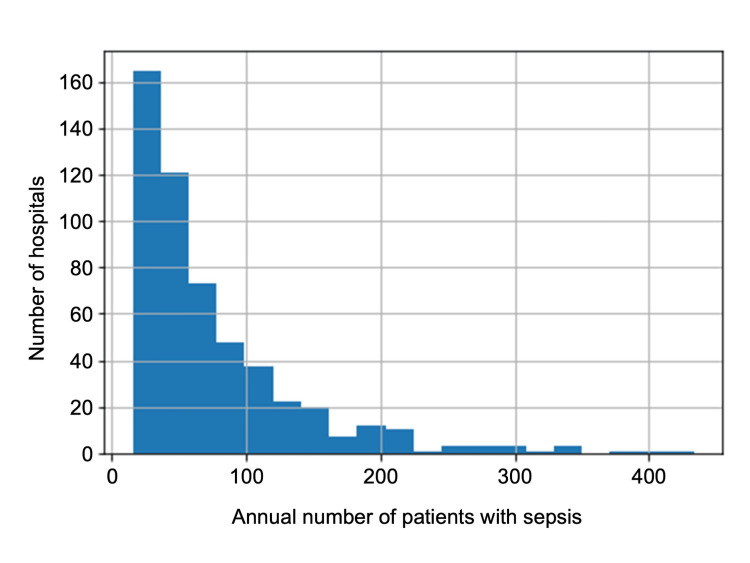
Histogram representation of hospitals according to the annual number of sepsis patients admitted to the intensive care unit. The histogram depicts the relationship between the number of sepsis patients admitted to the intensive care unit per hospital on the X-axis and the number of hospitals on the Y-axis.

During our study period, among the 50,490,128 registered patients on the DPC system from 532 hospitals, the data of 317,365 sepsis patients were included in this study (Figure 1). According to the annual number of admitted patients per institution, the ICU case volumes were categorized into four quartiles in ascending order as follows: Q1, Q2, Q3, and Q4 (Figure 2).

There were significant differences in age, sex, chronic diseases, percentage of community-acquired sepsis, and infection sites among the four quartiles. Q4 showed a higher proportion of patients with vasopressor therapy, hepatic disorder, and thrombocytopenia/coagulopathy. Additionally, Q4 had a higher number of hospital beds and a higher percentage of academic affiliations (Table 2).

**Table 2:** Baseline characteristics of the patients in the cohort. ^a^: Q1: 1–57 patients per year; Q2: 58–103 patients per year; Q3: 104–179 patients per year; and Q4: 179–434 patients per year. ^b^: Number of patients missing Barthel Index: Q1 32,131, Q2 33,634, Q3 34,798, Q4 34, 413. ICU = intensive care unit; IQR = interquartile range; RRT = renal replacement therapy

	Total^a^ (n = 317,365)	ICU case volume Q1 (n = 79,072)	ICU case volume Q2 (n = 79,027)	ICU case volume Q3 (n = 78,960)	ICU case volume Q4 (n = 80,306)
Number of patients with sepsis (n/year)	103 (58–180)	39 (30–48)	79 (68–91)	135 (116–151)	229 (198–318)
Age, years	73 (63–80)	74 (65–81)	73 (64–81)	72 (62–80)	71 (61–79)
Male, n (%)	200,773 (63.3)	50,013 (63.2)	49,628 (62.8)	50,112 (63.5)	51,020 (63.5)
Chronic diseases					
Cancer, n (%)	85,313 (26.9)	22,943 (29.0)	22,884 (29.0)	18,901 (23.9)	20,585 (25.6)
Hypertension, n (%)	91,901 (29.0)	22,711 (28.7)	21,982 (27.8)	23,760 (30.1)	23,449 (29.2)
Diabetes mellitus, n (%)	74,726 (23.5)	19,043 (24.1)	18,853 (23.9)	18,537 (23.5)	18,293 (22.48)
Heart failure, n (%)	81,521 (25.7)	20,854 (26.4)	20,147 (25.5)	21,327 (27.0)	19,194 (23.9)
Stroke, n (%)	51,334 (16.2)	13,403 (17.10)	12,796 (16.2)	13,040 (16.5)	12,095 (15.1)
Ischemic heart disease, n (%)	59,876 (18.9)	15,068 (19.1)	14,626 (18.5)	15,920 (20.2)	14,263 (17.8)
Chronic respiratory disease, n (%)	19,264 (6.1)	5,244 (6.6)	4,860 (6.1)	4,578 (5.8)	4,582 (5.7)
Chronic renal failure, n (%)	16,655 (5.2)	3,765 (4.8)	4,135 (5.2)	4,333 (5.5)	4,423 (5.5)
Barthel Index^b^					
0–60	71,937 (22.7)	18,994 (24.0)	17,106 (21.6)	19,146 (24.2)	16,691 (20.8)
61–99	19,839 (6.3)	5,324 (6.9)	5,365 (6.8)	5,081 (6.4)	4,069 (5.1)
100	90,613 (28.6)	22,623 (28.6)	17,106 (21.6)	19,935 (25.2)	25,133 (31.3)
Community-acquired sepsis, n (%)	125,124 (39.4)	32,617 (41.2)	32,123 (40.6)	31,285 (39.6)	29,099 (36.2)
Infection site					
Respiratory, n (%)	48,808 (15.4)	12,778 (16.2)	12,465 (15.8)	11,910 (15.1)	11,655 (14.5)
Abdominal, n (%)	30,009 (9.5)	8,210 (10.4)	7,994 (10.1)	7,055 (8.9)	6,750 (8.4)
Urogenital, n (%)	7,869 (2.5)	2,058 (2.6)	1,873 (2.4)	1,993 (2.5)	1,945 (2.4)
Bone and soft tissue, n (%)	5,828 (1.8)	1,411 (1.8)	1,475 (1.9)	1,414 (1.8)	1,528 (1.9)
Meninges/Brain/Spinal cord, n (%)	3,949 (1.2)	921 (1.2)	840 (1.1)	1,104 (1.4)	1,084 (1.3)
Heart, n (%)	3,975 (1.3)	982 (1.2)	891 (1.1)	1,142 (1.4)	960 (1.2)
Blood, n (%)	91 (0.03)	18 (0.02)	24 (0.03)	26 (0.03)	23 (0.03)
Multiple, n (%)	55,385 (17.5)	14,101 (17.8)	14,432 (18.3)	13,352 (16.9)	13,500 (16.8)
Unknown, n (%)	161,451 (50.9)	38,593 (48.8)	39,033 (49.4)	40,964 (51.9)	42,861 (53.4)
Vasopressor therapy, n (%)	94,271 (29.7)	22,551 (28.5)	23,988 (30.4)	23,100 (29.3)	24,632 (30.7)
Vasopressor therapy at the time of sepsis onset, n (%)	69,722 (22.0)	15,929 (20.1)	17,386 (22.0)	17,458 (22.1)	18,950 (23.6)
Oxygen therapy, n (%)	278,051 (87.6)	70,214 (88.8)	69,652 (88.1)	69,329 (87.8)	68,856 (85.7)
Oxygen therapy at the time of sepsis onset, n (%)	119,088 (37.5)	29,076 (36.8)	29,957 (37.9)	30,299 (38.4)	29,757 (37.1)
Mechanical ventilation, n (%)	186,720 (58.8)	43,936 (55.6)	46,838 (59.3)	48,902 (61.9)	47,044 (58.6)
Mechanical ventilation at the time of sepsis onset, n (%)	107,647 (33.9)	23,628 (29.9)	26,283 (33.3)	28,785 (36.5)	28,952 (36.1)
Kidney dysfunction, n (%)	196,711 (62.0)	48,739 (61.6)	48,708 (61.6)	50,823 (64.4)	48,441 (60.3)
RRT, n (%)	69,134 (21.8)	16,050 (20.3)	17,707 (22.4)	17,756 (22.5)	17,6321 (21.9)
RRT at the time of sepsis onset, n (%)	31,945 (10.1)	7,646 (9.7)	8,458 (10.7)	8,230 (10.4)	7,612 (9.5)
Hepatic disorder, n (%)	15,290 (4.8)	3,546 (4.5)	3,799 (4.8)	3,761 (4.8)	4,184 (5.2)
Thrombocytopenia/Coagulopathy, n (%)	63,620 (20.0)	15,704 (19.9)	16,175 (20.5)	15,074 (19.1)	16,668 (20.8)
Acidosis, n (%)	10,267 (3.2)	2,455 (3.1)	2,414 (3.1)	2,813 (3.6)	2,585 (3.2)
Total number of hospital beds					
≤272 beds	37,386 (11.8)	25,687 (32.5)	8,817 (11.2)	2,405 (3.0)	477 (0.6)
273–404 beds	67,407 (21.2)	29,089 (36.8)	22,254 (28.2)	13,486 (17.1)	2,578 (3.2)
405–587 beds	91,309 (28.8)	13,655 (17.3)	32,834 (41.5)	31,281 (39.6)	13,539 (16.9)
≥ 588 beds	121,263 (38.2)	10,641 (13.5)	15,122 (19.1)	31,788 (40.3)	63,712 (79.3)
Academic affiliation, n (%)	91,582 (28.9)	3,334 (4.2)	14,717 (18.6)	24,124 (30.6)	49,407 (61.5)

Clinical outcomes and medical costs

In-hospital mortality was 24.4% in the cohort, with the highest mortality recorded in Q1 (26.0%) and the lowest in Q4 (21.4%). The length of hospital stay was shorter in Q4 (35, range = 20-62) than in Q1 (42, range = 23-72). Gross medical costs were higher in Q4 ($2.86 × 10^10^) than in the other quartiles ($2.58 × 10^9^ in Q1, $2.67 × 10^9^ in Q2, and $2.78 × 10^9^ in Q3). The median cost per hospitalization and median daily costs per person increased proportionally with the hospital volume (Table 3).

**Table 3:** Clinical outcomes and medical costs in the cohort. ^a^: Q1: 1–57 patients per year; Q2: 58–103 patients per year; Q3: 104–179 patients per year; and Q4: 179–434 patients per year. ICU = intensive care unit; SD = standard deviation; IQR = interquartile range

	Total^a^ (n = 317,365)	ICU case volume Q1 (n = 79,072)	ICU case volume Q2 (n = 79,027)	ICU case volume Q3 (n = 78,960)	ICU case volume Q4 (n = 80,306)
In-hospital mortality, n (%)	77,405 (24.4)	20,546 (26.0)	20,790 (26.3)	18,866 (23.9)	17,204 (21.4)
Ventilator-free days					
Mean (SD)	21.3 (9.9)	21.5 (9.9)	21.3 (10.0)	21.1 (9.9)	21.3 (9.8)
Median (IQR)	27 (19–28)	27 (19–28)	27 (19–28)	26 (18–28)	27 (19–28)
Vasopressor-free days					
Mean (SD)	25.1 (7.8)	25.1 (7.8)	24.9 (8.0)	25.0 (7.9)	25.2 (7.6)
Median (IQR)	28 (27–28)	28 (27–28)	28 (27–28)	28 (27–28)	28 (27–28)
Length of hospital stay (days)					
Mean (SD)	55.0 (73.4)	58.5 (78.5)	56.4 (77.7)	53.7 (73.3)	51.2 (62.8)
Median (IQR)	39 (21–67)	42 (23–72)	40 (22–69)	38 (21–66)	35 (20–62)
Length of ICU stay (days)					
Mean (SD)	6.8 (6.2)	6.1 (5.1)	6.3 (5.4)	6.7 (6.0)	7.9 (7.2)
Median (IQR)	5 (2–11)	4 (2–10)	5 (2–10)	5 (2–11)	6 (2–13)
Length of antibiotic treatment (days)					
Mean (SD)	24.2 (28.4)	23.5 (26.0)	24.3 (28.3)	24.6 (29.3)	24.5 (29.8)
Median (IQR)	16 (9–29)	15 (9–28)	16 (9–29)	16 (9–29)	15 (9–29)
Inter-hospital transfer, % (n)	29.4 (93,304)	26.3 (20,776)	28.0 (22,138)	30.7 (24,203)	32.6 (26,187)
Gross medical costs ($)	1.09 × 10^10^	2.58 × 10^9^	2.67 × 10^9^	2.78 × 10^9^	2.86 × 10^10^
Medical costs per hospitalization ($)					
Mean (SD)	34,324 (34,939)	32,688 (30,024)	33,791 (33,563)	35,221 (36,363)	35,579 (39,021)
Median (IQR)	25,059 (14,401– 43,333)	24,305 (14,332– 41,815)	24,744 (14,339– 42,548)	25,757 (14,459–44,719)	25,537 (14,482– 44,208)
Daily medical costs per person ($)					
Mean (SD)	836 (823)	750 (732)	804 (804)	870 (846)	919 (889)
Median (IQR)	614 (443–953)	557 (411–836)	593 (431–905)	636 (455–999)	683 (482–1,064)

Regression analysis

After adjustment for baseline imbalances, the highest ICU case volume was significantly associated with reduced in-hospital mortality compared to the lowest hospital case volume (odds ratio (OR) = 0.829, 95% confidence interval (CI) = 0.794-0.865; p < 0.001). Additionally, in-hospital mortality in Q3 was lower than that in Q1 (OR = 0.929, 95% CI = 0.898-0.961; p < 0.0001) (Table 4).

**Table 4:** Multivariable regression analysis for in-hospital mortality. ^a^: Q1: 1–57 patients per year; Q2: 58–103 patients per year; Q3: 104–179 patients per year; and Q4: 179–434 patients per year. ^b^: The independent variable was adjusted for age, sex, site of infection, community-acquired sepsis, chronic diseases, Barthel Index, vasopressor therapy at the time of sepsis onset, mechanical ventilation at the time of sepsis onset, renal replacement therapy at the time of sepsis onset, department, admission year, number of hospital beds, and academic affiliation. CI = confidence interval; ICU = intensive care unit; IQR = interquartile range

ICU case volume categories^a, b^	Odds ratio	95% CI	P-value
Q1	1.000	Reference	
Q2	1.030	0.999–1.062	0.056
Q3	0.929	0.898–0.961	<0.001
Q4	0.829	0.794–0.865	<0.001

**Figure 3:**
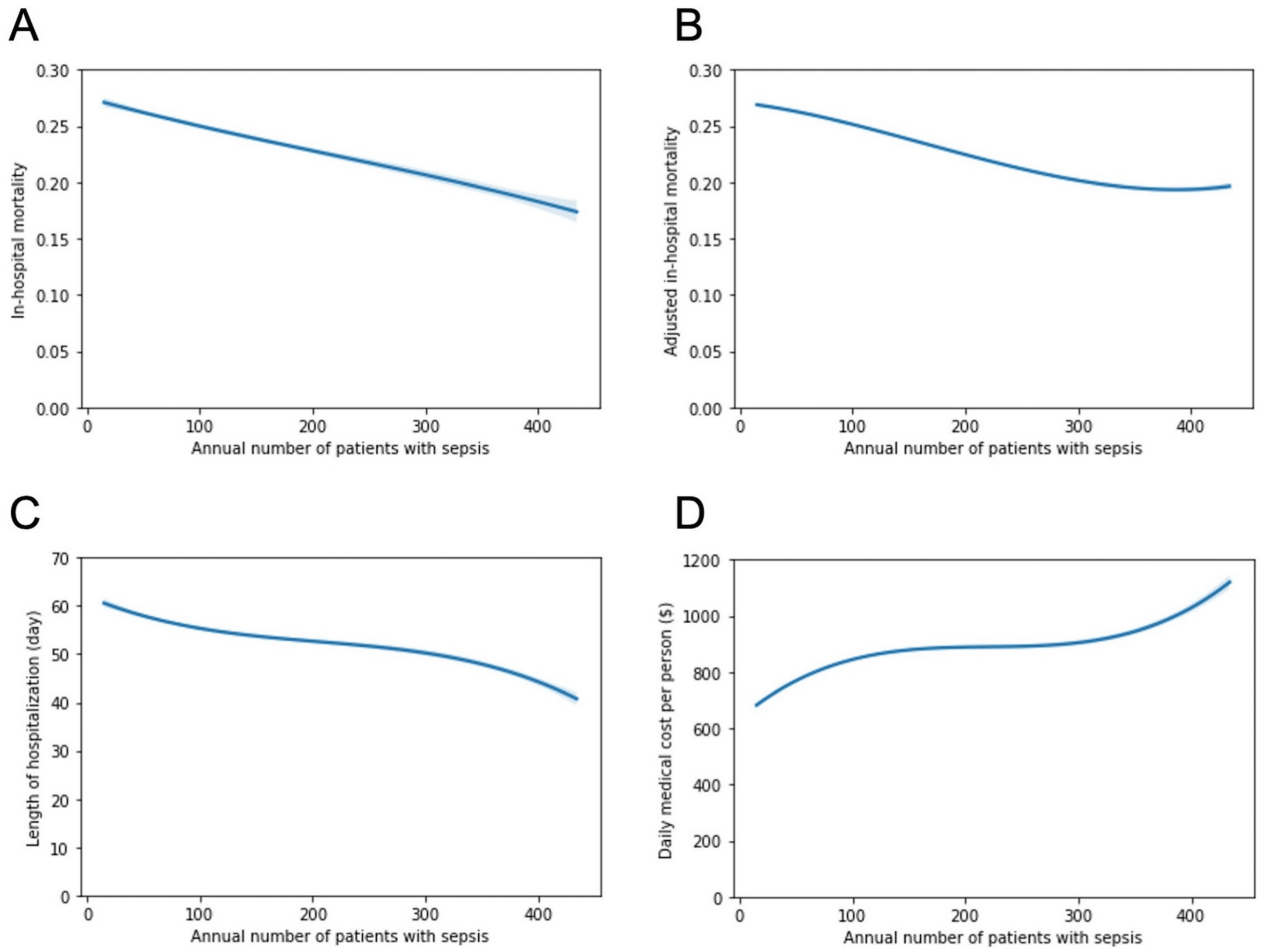
Association between the annual number of patients with sepsis and in-hospital mortality, length of hospital stay, and medical costs. The polynomial regression plot shows the relationship between the log-transformed annual number of sepsis patients per hospital on the X-axis and the (A) unadjusted in-hospital mortality, (B) adjusted in-hospital mortality, (C) length of hospital stay, and (D) daily medical costs per person, respectively. The line depicts the trinomial regression according to the parameters with the 95% confidence interval.

Fractional polynomials exhibited that the annual number of patients per institution was significantly associated with reduced in-hospital mortality. While the length of hospital stay decreased in proportion to the sepsis case volume, the daily medical costs per person significantly increased with the increase in the case volume (Figure 3).

Subgroup analysis

**Figure 4:**
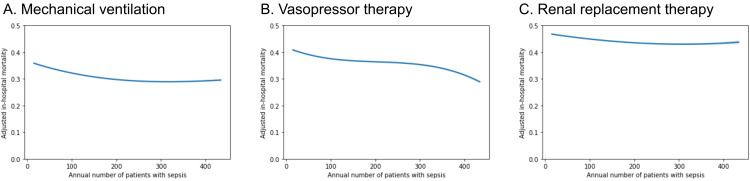
Subgroup analyses of the effect of intensive care unit case volume on in-hospital mortality. The polynomial regression plot shows the relationship between the log-transformed annual number of sepsis patients per hospital on the X-axis and in-hospital mortality on the Y-axis in patients subjected to (A) mechanical ventilation, (B) vasopressor therapy, and (C) renal replacement therapy. The line depicts the trinomial regression according to the parameters with the 95% confidence interval.

In the subpopulation subjected to mechanical ventilation, vasopressor therapy, and RRT, the annual number of sepsis patients was significantly associated with reduced in-hospital mortality (Figure 4).

Sensitivity analysis

**Figure 5:**
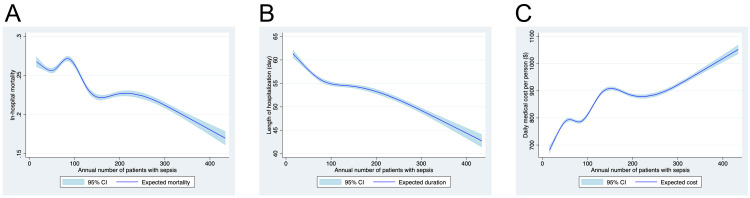
Association between the annual number of sepsis patients and in-hospital mortality using restricted cubic splines. The cubic spline restriction plot shows the relationship between the annual number of sepsis patients per hospital on the X-axis and the (A) in-hospital mortality (six knots), (B) length of hospital stay (four knots), and (C) daily medical cost per person (six knots) on the Y-axis, respectively. The line depicts the trinomial regression according to the parameters with the 95% confidence interval (CI).

Restricted cubic splines showed similar correlation patterns as that of fractional polynomials between the annual number of patients per institution and in-hospital mortality (six knots), length of hospital stay (four knots), or the daily medical costs per person (six knots) (Figure 5, Table 5). After excluding patients who were transferred to other hospitals, Q4 still showed lower in-hospital mortality than that of Q1 (OR = 0.828, 95% CI = 0.811-0.845), which was consistent with the primary test results (Table 6). Moreover, after applying the threshold for outliers with 5% of top and bottom number of sepsis cases, in-hospital mortality in Q4 was significantly lower than that in Q1 (OR = 0.886, 95% CI = 0.845-0.930) (Table 7).

**Table 5:** Multivariable regression analysis for in-hospital mortality after exclusion of hospitals with the top and bottom 5% of the annual number of patients with sepsis. ^a^: Q1: 1–60 patients per year; Q2: 61–103 patients per year; Q3: 104–174 patients per year; and Q4: 175–344 patients per year. ^b^: The independent variable was adjusted for age, sex, site of infection, community-acquired sepsis, chronic diseases, Barthel Index, vasopressor therapy at the time of sepsis onset, mechanical ventilation at the time of sepsis onset, renal replacement therapy at the time of sepsis onset, department, admission year, number of hospital beds, and academic affiliation. ICU = intensive care unit; CI = confidence interval

ICU case volume categories^a, b^	Odds ratio	95% CI
Q1	1.000	Reference
Q2	1.042	1.007–1.079
Q3	0.979	0.942–1.017
Q4	0.886	0.845–0.930

**Table 6:** A comparison of the log-likelihood and information criteria for the restricted cubic spline models. df = degree of freedom; AIC = Akaike’s information criterion; BIC = Bayesian information criterion

Model	df	AIC	BIC
In-hospital mortality			
3 knots	317,364	363,368	363,400
4 knots	317,364	363,330	363,372
5 knots	317,364	363,330	363,384
6 knots	317,364	363,214	363,278
Length of hospitalization			
3 knots	317,364	3,626,434	3,626,466
4 knots	317,364	3,626,395	3,626,438
5 knots	317,364	3,626,394	3,626,447
6 knots	317,364	3,626,389	3,626,453
Daily medical cost per person ($)			
3 knots	317,364	5,159,088	5,159,120
4 knots	317,364	5,159,097	5,159,140
5 knots	317,364	5,159,083	5,159,136
6 knots	317,364	5,158,844	5,158,908

**Table 7:** Multivariable regression analysis for in-hospital mortality after the exclusion of transferred patients. ^a^: Q1: 1–57 patients per year; Q2: 58–103 patients per year; Q3: 104–179 patients per year; and Q4: 179–434 patients per year. ^b^: The independent variable was adjusted for age, sex, site of infection, community-acquired sepsis, chronic diseases, Barthel Index, vasopressor therapy at the time of sepsis onset, mechanical ventilation at the time of sepsis onset, renal replacement therapy at the time of sepsis onset, department, admission year, number of hospital beds, and academic affiliation. ICU = intensive care unit; CI = confidence interval

ICU case volume categories^a, b^	Odds ratio	95% CI
Q1	1.000	Reference
Q2	1.042	1.007–1.079
Q3	0.979	0.942–1.017
Q4	0.886	0.845–0.930

## Discussion

Using the Japanese nationwide medical claims database, this study demonstrated that a high ICU case volume was significantly associated with lower in-hospital mortality in patients with sepsis. Moreover, high-volume ICUs had higher daily medical costs per person in sepsis than low-volume ICUs. Furthermore, a higher ICU sepsis caseload was significantly associated with lower in-hospital mortality in patients receiving mechanical ventilation, vasopressor therapy, and RRT.

The discourse on the relationship between ICU case volume and mortality in patients with sepsis has been complex and multifaceted, with studies supporting both sides of the argument regarding the impact of case volume on mortality [8-13]. The disparities in findings may be due to differences in study design, sample size, adjusted variables, and the unique healthcare systems across different countries. Of the six studies focusing on this relationship, one enrolled only patients with malignancy [9], and another included only patients with septic shock [12]. Our discussion extends into a relatively unexplored area of research from Asia, particularly Japan, highlighting a critical gap that indicates potential differences in medical practices or outcomes among Asian populations. It is particularly relevant to the Japanese healthcare context, and potentially to other Asian healthcare systems, where such detailed analyses are sparse. This work paves the way for a better understanding of how hospital capacity and patient volume influence health outcomes and resource allocation in critical care, particularly in sepsis management.

Additionally, the debate on the optimal size and case volume of ICUs is ongoing, and as more evidence emerges to support the critical role of ICUs [23], our study contributes to this discussion by identifying a volume threshold. Previous studies have indicated various thresholds, but our research found that an annual case volume of 104 patients with sepsis was associated with improved outcomes. This is in contrast to the UK results suggesting a volume threshold of 215 cases per year [11] and is more consistent with studies suggesting a lower threshold of 40 cases of septic shock per year [12]. Our study adds significant weight to this debate, with a larger sample size than previous studies, thereby enhancing the reliability of our results. This substantial sample size is a major strength of our study, which is likely to provide a more conclusive answer regarding the impact of hospital case volume on mortality and medical costs in sepsis.

The mechanism of the beneficial effect of high-volume ICUs on mortality is potentially explained by several components, including abundant medical resources, more experienced medical staff, and higher capability of implementing evidence-based practices [27]. It seems reasonable that the availability of abundant medical resources with more experienced staff contributes to improving the clinical outcomes of sepsis patients. On the other hand, considering that patients subjected to vasopressor therapy, mechanical ventilation, or RRT comprised fewer than 60% of the cohort, these components may not substantially affect the benefits of high-volume hospitals. As the intensity of physician staffing and compliance with implementing guidelines-based treatment were not investigated in our study, detailed mechanisms of the effect of ICU case volume on mortality should be clarified in future investigations.

Although we demonstrated higher daily medical costs per person and shorter length of stay in association with higher sepsis case volume, previous publications rarely focused on economic outcomes [28]. The novel angle of healthcare costs concerning ICU case volume adds a fresh perspective to the existing literature, which has largely focused on clinical outcomes. Besides sepsis, high-volume hospitals were associated with better survival and lower total cost per admission for severe trauma patients [15]. In contrast, a previous study enrolling burn injury patients demonstrated a positive correlation between the hospital case volume and medical costs during hospitalization [16]. The plausible reason for the increased medical costs was the high proportion of intensive treatments performed in high-volume hospitals, such as mechanical ventilation and cultured skin grafts. Our study demonstrated that not only daily medical costs per person but also medical costs per hospitalization increased according to the number of annual sepsis patients, despite a shorter length of stay. As the proportion of patients on artificial organ support is greater in high-volume ICUs than in low-volume ICUs, the proportional association between the sepsis case volume and medical costs would be conceivable.

ICU case volume was shown to be a crucial factor in improving in-hospital mortality in this study; however, centralization of sepsis patients requires deep consideration and meticulous debate before policy implementation. While the acceleration of the centralization improved the survival rate after pancreatic cancer surgery [29], it might not be appropriate to apply such a policy to sepsis management without careful deliberation. As sepsis patients require a time-sensitive approach with early recognition and immediate initiation of treatment, transportation of sepsis patients could reduce the treatment effect due to delayed antibiotic administration and the introduction of sepsis management bundles [30]. Therefore, investigation of the detailed mechanisms responsible for better clinical outcomes in high-volume ICUs should be conducted rather than encouraging the transportation of sepsis patients to high-volume ICUs.

This study has several limitations. First, the administrative database did not include laboratory data to calculate the severity scores such as the sequential organ failure assessment scores. Second, despite an established method for identifying patients with sepsis in the administrative data, the risk of misclassification may lead to over- or underestimation of sepsis cases; however, all individuals required ICU admission, which at least ensured the target population of serious infection with critical illness in this study. Third, treatment policy such as withholding or withdrawal of life-sustaining treatment was not collected from the database, which could be a confounding factor affecting the results. Fourth, patients transferred from other hospitals were not distinguished from other patients. Therefore, such a population could lead to a selection bias. Fifth, long-term mortality rate and quality of life were not investigated in the cohort. Sixth, plausible reasons for the association between the hospital case volume and the mortality or medical costs were not sufficiently explained. Further research, including a prospective study and external validation, is warranted to verify the current findings and elucidate the mechanisms underlying these intimate relationships before encouraging the centralization of sepsis patients.

## Conclusions

This study, using a Japanese nationwide medical claims database, suggested that higher-volume ICUs may be associated with better survival and higher daily medical costs per person in sepsis cases compared to lower-volume ICUs. Additionally, high-volume ICUs with patients receiving mechanical ventilation, vasopressor therapy, and RRT exhibited significantly lower in-hospital mortality. Further research is warranted to validate these results and clarify the mechanisms underlying the ICU case volume-outcome relationship in patients with sepsis.
